# Preparation and Performance of Silicone Rubber Composites Modified by Polyurethane

**DOI:** 10.3390/polym15193920

**Published:** 2023-09-28

**Authors:** Lijun Gao, Ying Li, Wensheng Fu, Liming Zhou, Shaoming Fang

**Affiliations:** Henan Provincial Key Laboratory of Surface & Interface Science, School of Material and Chemical Engineering, Zhengzhou University of Light Industry, Zhengzhou 450002, Chinazlming1212@126.com (L.Z.)

**Keywords:** silicone rubber, polyurethane, composite, interpenetrating polymer network, mechanical property

## Abstract

Silicone rubber composites with good comprehensive properties modified with polyurethane were obtained through mixing and vulcanizing methods. Firstly, the polyurethane prepolymer with double bonds was prepared by polytetrahydrofuran glycol (PTMG, Mn = 1000), isophorone diisocyanate (IPDI), and 2-hydroxyethyl methacrylate (HEMA). The prepolymer was then added to the silicone rubber compounds to prepare silicone rubber composites, combining the excellent properties of polyurethane with the silicone rubber materials. The effects of polyurethane content on the mechanical properties, insulation, hydrophobicity, thermal stability, and flame retardancy of composites were studied in detail. The results showed that the silicone rubber composites not only have good hydrophobicity, thermal stability and flame retardant properties, but the addition of polyurethane significantly improves the tensile strength at room and low temperatures and the volume resistivity of the materials. The tensile strength increased by 32.5%, and the volume resistivity nearly doubled. The excellent electrical insulation, high hydrophobicity and good mechanical properties make the silicone rubber composites appropriate for use in the field of polymeric house arresters.

## 1. Introduction

In the chemical structure of silicone rubber (SIR), the Si-O bonds provide thermal stability, weather and oxidation resistance. In addition, due to the small amount of surface energy, silicone insulation materials have the characteristics of organic and inorganic materials. In recent years, insulation materials improved by silicone rubber have been widely used in high-voltage outdoor insulators, such as overhead high-voltage transmission lines, transformers and reactors [1]. Compared with traditional ceramic or glass high-voltage insulators, silicone rubber composite insulators have the characteristics of being lightweight, easy to maintain and resistant to pollution. By adding different types of fillers to silicone resin, the electrical, mechanical and thermal properties of silicone resin can be further improved to meet various requirements of outdoor applications [2,3].

SIR used as outdoor insulation materials need to have high hydrophobic and antifouling properties. The excellent waterproof performance of a silicon rubber insulator is attributed to the diffusion of low molecular weight siloxane chains (methyl vinyl silicone rubber) from the bulk of the material to its surface, thus forming a lattice-type hydrophobic thin layer [4]. In a severely to moderately polluted environment, the formation of a water layer on an insulator surface is avoided, the formation of a leakage current is reduced, and flashover is avoided [5,6,7]. The change in contact angle of superhydrophobic silicone rubber after long-term contact with water is a necessary condition to consider the outdoor durability of superhydrophobic silicone rubber. Magnesium oxide, zinc oxide and carbon tetrafluoride (CF_4_) can be used as fillers to elevate the hydrophobicity of silicone rubber insulation materials [8,9].

SIR used for outdoor insulation materials also needs to have high resistivity, thermal conductivity and electrical strength. Adding metal oxides, nitrides and reduced graphene oxide can increase the insulation’s performance [10,11,12,13]. In order to improve the electrical properties, the erosion and wear resistance of silicone rubber in the field of high-voltage insulation, SIR composites were prepared by filling them with aluminum hydroxide (ATH), irregular silica (IS), spherical silica (SS) and silicon dioxide (SiO_2_) by mechanical blending [14,15,16]. Zhang et al. adopted the modification method of coating polydopamine (PDA) on the surface of hexagonal boron nitride (h-BN) to prepare composite materials based on h-BN@PDA and silicone rubber with good thermal conductivity and high volume resistivity [17]. Coincidentally, Khanum et al. suggested that adding a small amount of h-BN nanosheets to silica–siloxane composites significantly improved the thermal conductivity and corrosion resistance of the materials, and even at a high temperature, it would not change the dielectric response of these hybrid composites [18].

As an outdoor insulating material, the excellent mechanical properties of SIR are helpful to improve the service life of the material. Amine-containing silicone resin (A-MQ) can enhance the tensile strength and elongation at the break of silicone rubber (ALSR) [19]. As SIR fillers, SiO_2_, alumina, silicon carbide, graphite oxide and carbon nanotubes can improve the mechanical properties and insulation of materials [20,21,22].

In the modification of various silicone rubber insulator composites, most of them are modified by inorganic nano-fillers, but few are modified by polymer interpenetrating networks. Therefore, in this paper, polyurethane is used to modify silicone rubber to form an interpenetrating polymer network so that the composites not only have the excellent performance of silicone rubber but also have the characteristics of corrosion resistance and the high mechanical strength of polyurethane. Firstly, a polyurethane prepolymer with double bonds was synthesized. Then, the synthesized prepolymer was added to silicone rubber, and a series of silicone rubber resins were prepared by adjusting the content of the polyurethane prepolymer. When the silicone rubber and polyurethane prepolymer were blended and heated, they underwent a cross-linking reaction to form an interpenetrating polymer network structure. The effect of the polyurethane content on the comprehensive properties of the material was studied in detail.

## 2. Materials and Methods

### 2.1. Materials

Methyl silicone rubber was provided by Zhejiang Hesheng Silicon Industry Co., Ltd. (Pinghu, China). White carbon black (amorphous silica) was provided by Jiangxi Kehong New Material Technology Co., Ltd. (Yichun, China). Aluminum hydroxide was provided by Tianjin Comio Chemical Reagent Co., Ltd. (Tianjin, China). Hydroxyl-terminated polydimethylsiloxane, maleic anhydride graft copolymer, antioxidant 1010, 2,5-dimethyl-2,5-bis(tert-butyl peroxide) hexane, polytetrahydrofuran glycol (PTMG, Mn = 1000), isophorone diisocyanate (IPDI), dibutyltin laurate (DBTL), 2-hydroxyethyl methacrylate (HEMA) and 2,2′-azobis(2-methylpropionitrile) (AIBN) were provided by Shanghai Macklin Biochemical Technology Co., Ltd. (Shanghai, China).

### 2.2. Synthesis of Polyurethane Prepolymer

Amounts of 50.000 g PTMG and 22.228 g IPDI were added into a three-necked flask and stirred at 40 °C for 15–30 min. Then, 0.086 g DBTL was added, and after 60 min of constant temperature stirring, 13.014 g HEMA and 0.256 g AIBN were added, and the reaction continued for 30 min. Finally, the polyurethane prepolymer was obtained and stored in a dry bottle for later use.

### 2.3. Preparation of Silicone Rubber Composites

The reparation of silicone rubber composites with 0–10 phr (“phr” is the abbreviation for “parts per hundred parts of resin”, representing the amount to be added to every 100 parts of silicone rubber matrix) of polyurethane is divided into two steps. The first step is to mix the materials evenly on a double-roller blending mill to obtain mixed rubber, and the second step is to vulcanize the mixed rubber on a plate vulcanization machine. The specific experimental steps are as follows.

Firstly, 100 phr methyl vinyl silicone rubber, 40 phr white carbon black, 2 phr hydroxyl-terminated polydimethylsiloxane and 2 phr maleic anhydride graft copolymer were added into the double-roller blending mill for mixing for about 15–20 min at 130 °C. Then, 1 phr antioxidant 1010 and 110 phr aluminum hydroxide were added. After the silicone rubber compound was uniformly plasticized, the compound was taken out for later use. Finally, the double-roller blending mill was cooled to 90 °C, and the obtained silicone rubber compound, 1–10 phr polyurethane prepolymer and 1 phr 2,5-dimethyl-2,5-bis(tert-butyl peroxide) hexane were added to the mill and mixed evenly to obtain the mixed rubber.

The mixed rubber was vulcanized at a high temperature on the plate vulcanizing machine at a pressure of 10 MPa. The vulcanization was divided into two time periods. The first vulcanization was molded at 180 °C for 5 min, then cold pressed for 3 min. The second vulcanization was performed at 180 °C for 30 min, then cold pressed for 3 min. Finally, a series of plate-shaped modified silicone rubbers was obtained.

The modified silicone rubber composites obtained by adding 0, 1, 2, 3, 4, 5, 7 and 10 phr polyurethane prepolymers were named MSR-0, MSR-1, MSR-2, MSR-3, MSR-4, MSR-5, MSR-7 and MSR-10, respectively.

### 2.4. Characterization

Infrared spectra were collected by the Fourier transform infrared (FTIR) spectrometer (Bruker TENSOR II, Karlsruhe, Germany). Each sample was scanned 16 times at a resolution of 0.5 cm^−1^. The extraction test was carried out according to GB/T18474-2001 [23]. The silicone rubber composites were soaked in acetone (a good solvent for polyurethane) and refluxed at 57 °C for 24 h. Finally, the weight of the composites was tested, and the weight loss rate was calculated. X-ray diffraction (XRD) patterns were tested by a Bruker D8 ADVANCE X-ray diffractometer (Karlsruhe, Germany). Cu Kα radiation was obtained from a copper X-ray tube operated at 40 kV and 30 mA. The measurement range of 2 theta was between 5° and 80°. The data were collected with an angular step of 0.05°. The viscosity of the sample was tested by the HAAKEMARSIII rotary shear rheometer of Thermo Fisher Scientific company (Waltham, MA, USA). The sample size was a disc with a diameter of 20 mm and a thickness of 2 mm, the test temperature was 30–180 °C, the number of steps was 20, the maximum waiting time was 60 s, and the heating rate was 1.25°/s. The water contact angles were tested using a contact angle meter (JC2000C, Shanghai Changzheng Electronic Technology Co., Ltd., Shanghai, China). The water absorption rate of the material was measured according to GB/T1034-2008, and the material was immersed in distilled water at 23 °C for 24 h and then weighed for calculation. The volume resistivity was conducted using an ultra-high resistance meter (ZC43, Nanjing Baifang Instrument Co., Ltd., Nanjing, China) with a charging time of 60 s and a charging voltage of 500 V. The tensile strength and elongation at break were performed using the electronic universal testing machine (Perkin Elmer, UTM2202, Waltham, MA, USA), where samples were prepared according to GB/T 1040.2-2006, with a thickness of 4 mm and the stretching rate of 200 mm/min. The hardness was measured with a shore durometer, and the model is LX-D-1 (Sichuan Huiju Instrument Equipment Co., Ltd., Luzhou, China). The thermostability was studied using a comprehensive thermal analyzer (Perkin Elmer, Diamond TG/DTA, Waltham, MA, USA) in a nitrogen atmosphere, and the nitrogen flow rate is 100 mL/min, the heating rate is 10 °C/min, the temperature range is 30–800 °C. According to GB/T2406, the limiting oxygen index of the materials was tested with an oxygen index meter (JL-JF-3, Beijing Beiguang Jingyi Instrument Equipment Co., Ltd., Beijing, China). The morphologies of silicone rubber composites were measured using a scanning electron microscope (SEM, JSM-7001F, JEOL Ltd., Tokyo, Japan), where the samples were broken by an electronic universal testing machine and plated with a layer of gold film.

## 3. Results and Discussion

### 3.1. Structure of Materials

**Figure 1:**
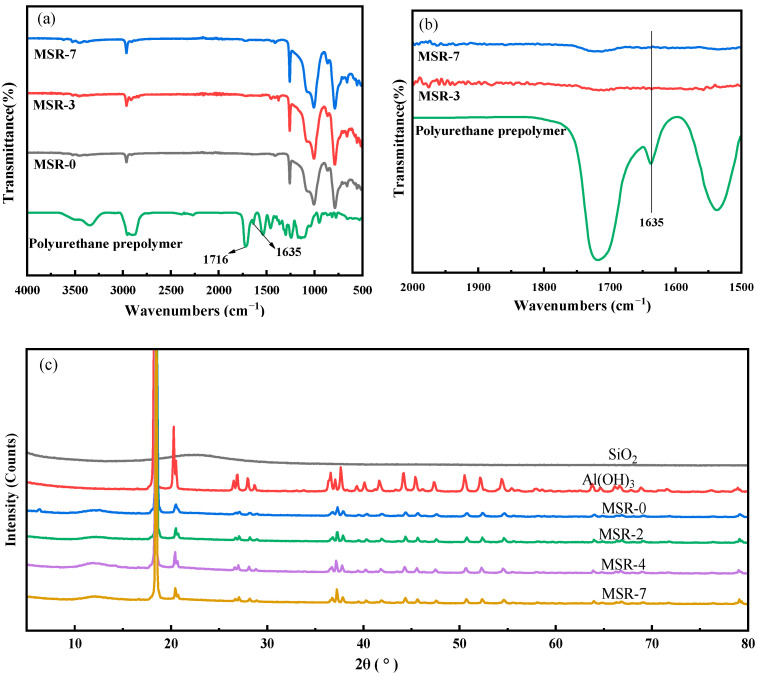
(**a**,**b**) Infrared spectra of polyurethane prepolymer and silicone rubber composites and (**c**) XRD patterns of SiO_2_, Al(OH)_3_, and silicone rubber composites.

As can be seen from the infrared spectra (Figure 1), there is no absorption peak of the isocyanate group (-NCO) in the polyurethane prepolymer at around 2200 cm^−1^, but the stretching vibration absorption peak of carbonyl (-C=O) on the carbamic ester group appears at 1716 cm^−1^, indicating that the reaction of isophorone diisocyanate is complete. In the infrared spectra of silicone rubber composites (MSR-3 and MSR-7), the absorption peak of the double bond (-C=C-) disappeared, which was originally 1635 cm^−1^ in the polyurethane prepolymer (Figure 1b), meaning that the double bond undergoes a reaction during the vulcanization process of silicone rubber.

In addition, it can also be seen from the results of the extraction experiment that the weight of the composites changes little before and after extraction (Table 1). As the solvent used is acetone, which is a good solvent for polyurethane, it indicates that polyurethane has undergone a cross-linking-curing reaction.

The XRD patterns of SiO_2_, Al(OH)_3_, and silicone rubber composites with different polyurethane contents are presented in Figure 1c. A broadened peak is observed in the SiO_2_, indicating the existence of amorphous silica. The composites have a wide peak at about 12°, which is the amorphous peak of silicone rubber. In addition, the other obvious strong diffraction peaks cannot be found in the composites except for the peaks of the Al(OH)_3_. The highly ordered crystal structure of Al(OH)_3_ was mostly preserved and not affected significantly in the silicone rubber matrix.

### 3.2. Viscosity Properties of Materials

**Figure 2:**
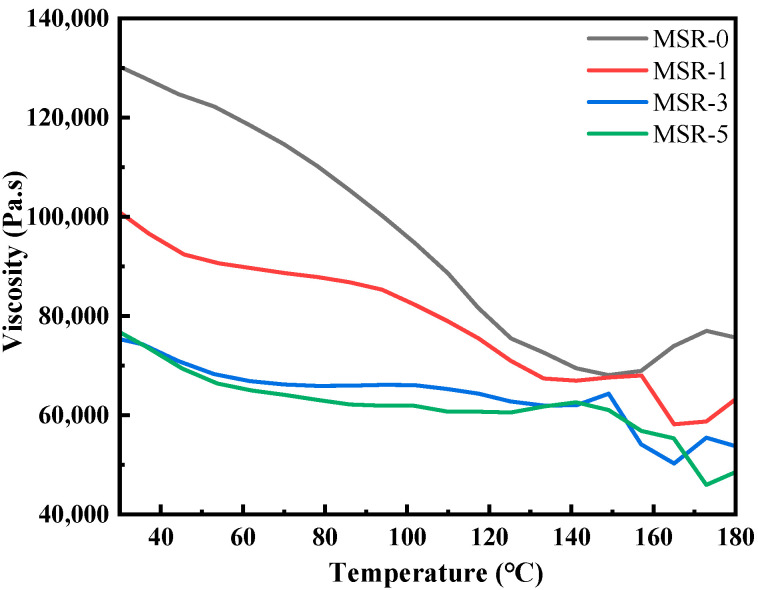
Effect of polyurethane content on the viscosity of silicone rubber composites.

Figure 2 shows the viscosity of silicone rubber composites as a function of temperature. In the temperature range of 30–140 °C, the viscosity of MSR-0 decreases significantly with the increase in temperature. The viscosity of MSR-0 at 30 °C is 130,000 Pa·s, which is reduced by nearly half at 140 °C. At the same temperature, with the increase in polyurethane content, the viscosity of the composites gradually decreases. Due to the addition of low-viscosity polyurethane prepolymers, the viscosity of silicone rubber composites is obviously lower than that of MSR-0 at 30 °C, and the viscosity of MSR-3 and MSR-5 decreases to around 75,000 Pa·s. When the temperature rises to the range of 155–172 °C, which has reached the vulcanization temperature of silicone rubber, the silicone rubber begins to cross-link curing, resulting in an increase in viscosity. It can be found that the viscosity of MSR-0, MSR-1, MSR-3 and MSR-5 begins to increase after the temperature rises to 155 °C, 165 °C, 165 °C and 172 °C, respectively. MSR-3 and MSR-5 exhibit a plateau at about 90 °C, which means that the viscosity does not decrease with the increase in temperature. This may be due to the phenomenon that the increase in viscosity caused by the polymerization of some polyurethane prepolymers offsets the decrease in viscosity caused by the increase in temperature.

### 3.3. Electrical Insulation Performance

**Figure 3:**
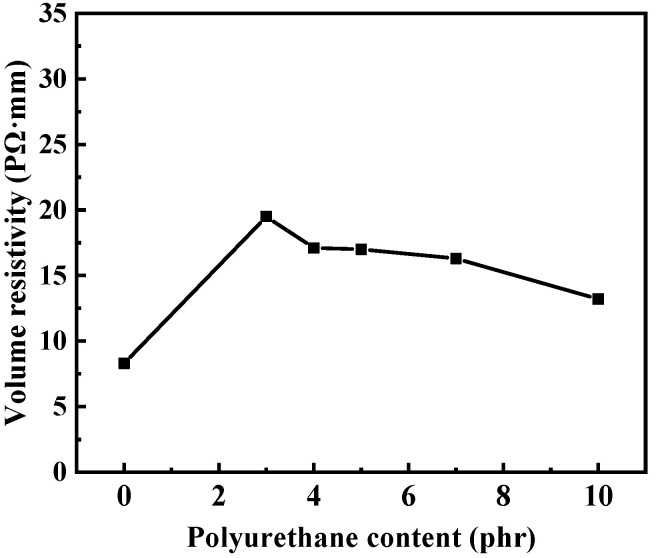
Effect of polyurethane content on the volume resistivity of silicone rubber composites.

The volume resistivity of silicone rubber composites is shown in Figure 3. The volume resistivity of the silicone rubber compound without polyurethane is 8.31 PΩ·mm. When the additional amount of polyurethane is 3, 4, 5, 7 and 10 phr, the volume resistivities of silicone rubber composites are 19.5, 17.1, 17.0, 16.3 and 13.2 PΩ·mm, respectively. Obviously, after the addition of polyurethane, the volume resistivities of the silicone rubber composites increase, indicating that the electrical insulation performance is enhanced. Due to the strong intermolecular force of polyurethane, the transfer and orientation of the carrier and polar group in the electric field are greatly restrained, resulting in a low degree of polar orientation, thereby increasing the volume resistivity.

### 3.4. Hydrophobic Properties of Materials

**Figure 4:**
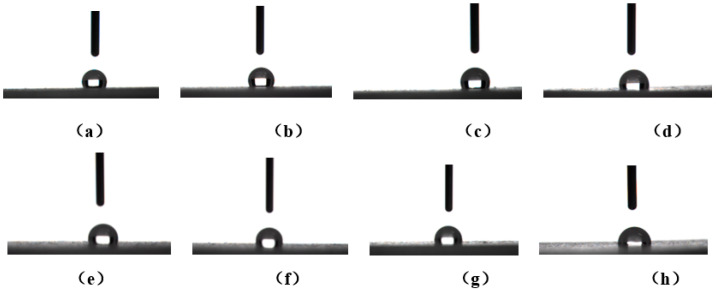
Droplet contact angle diagram on the surface of (**a**) MSR-0, (**b**) MSR-1, (**c**) MSR-2, (**d**) MSR-3, (**e**) MSR-4, (**f**) MSR-5, (**g**) MSR-7 and (**h**) MSR-10.

**Figure 5:**
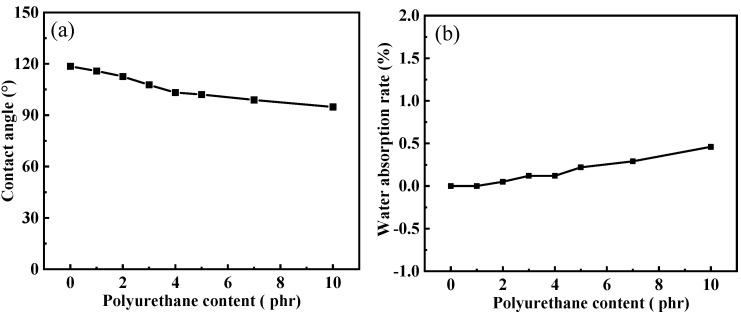
Effect of polyurethane content on (**a**) water contact angle and (**b**) water absorption rate of silicone rubber composites.

The hydrophobic properties and water contact angle of silicone rubber composites are shown in Figure 4 and Figure 5. The water contact angle of the composite without polyurethane is 118.5°. When polyurethane content is 1, 2, 3, 4, 5, 7 and 10 phr, the contact angles are 115.8°, 112.6°, 107.7°, 103.2°, 102.1°, 98.9° and 94.8°, respectively. The water contact angle of all materials is greater than 90 °, indicating good hydrophobicity. This may be due to the fact that the free micromolecule siloxane in the molecular network structure of silicone rubber can diffuse to the surface of the material, making its surface hydrophobicity [24,25]. However, silicone rubber composites have a slight decrease in water contact angle after adding polyurethane. The main chain of polyurethane contains carbamic ester and ether bonds, and the two groups have certain hydrophilicity. After blending with silicone rubber, hydrophilic groups are introduced into the material, which leads to a decrease in the hydrophobicity of the composites to a certain extent. As can be seen from Figure 5b, as predicted, the addition of polyurethane increases the water absorption rate of the composites. The smaller the water absorption of insulation materials, the better their insulation performance. The water absorption rate of insulation materials is usually required to be less than 0.40%. When the content of polyurethane is 4 phr, the water absorption rate of the material is 0.37%, which is lower than 0.40%. Therefore, when the content of polyurethane is equal to or lower than 4 phr, the composites meet the requirements for the water absorption of the insulation material. When the content of polyurethane exceeds 4 phr, the water absorption rate reaches 0.57–0.58%, which is greater than 0.40% and cannot meet the requirements of insulation materials. Therefore, when used as an insulation material, the content of polyurethane cannot exceed 4 phr.

### 3.5. Mechanical Property

**Figure 6:**
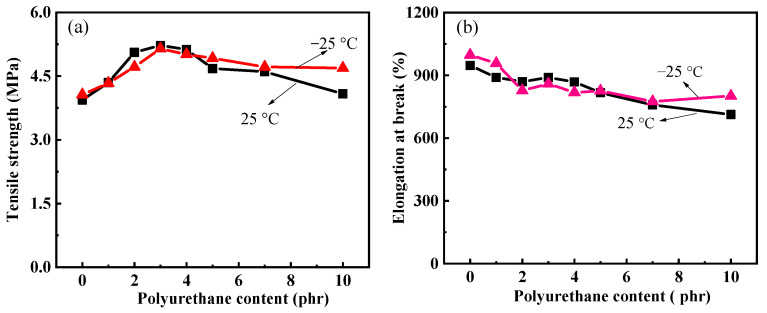
Effect of polyurethane content on the (**a**) tensile strength and (**b**) elongation at break of silicone rubber composites at room temperature (25 °C) and low temperature (−25 °C).

**Figure 7:**
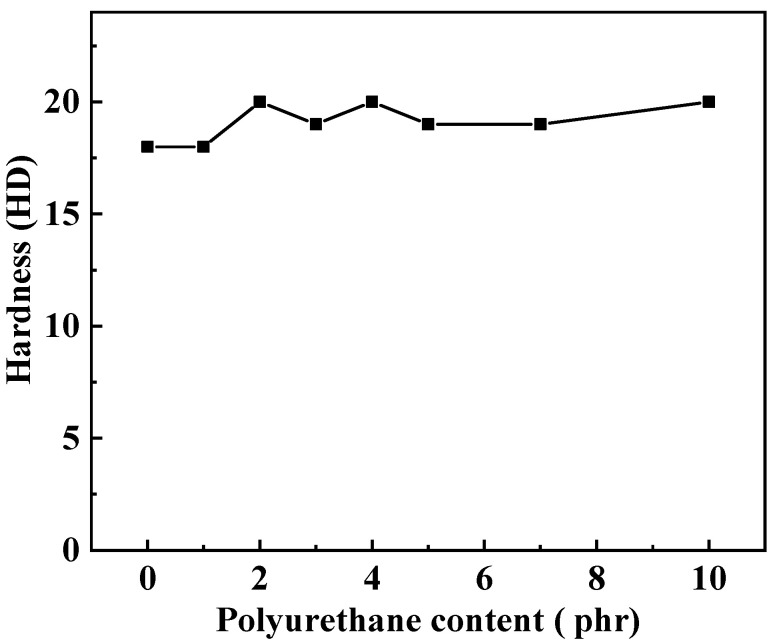
Effect of polyurethane content on hardness of silicone rubber composites.

The tensile strength and elongation at break of silicone rubber composites were tested at room temperature (25 °C) and low temperature (−25 °C), respectively. The results of the measurements are shown in Figure 6. At room temperature, with the increase in polyurethane content, the tensile strength of the composites shows a trend of first increasing and then decreasing. When the polyurethane content is 3 phr, the tensile strength of MSR-3 reaches the highest value of 5.22 MPa, which is 32.5% higher than that of MSR-0 without polyurethane. This indicates that there is a saturation value when polyurethane is used as a reinforcing agent in silicone rubber. However, the elongation at break shows different trends, which gradually decrease with the increase in polyurethane content. The polyurethane prepolymer has two double bonds at the end of the molecular chain, which react when heated. The interpenetrating polymer network [26] structure was formed after polyurethane and silicone rubber were crosslinked and cured. When subjected to external force, the polyurethane and silicone rubber molecules intertwine into a whole, forming a “forced compatibility” effect of the interpenetrating network, making it difficult for the molecular chains to disentangle, resulting in an increase in tensile strength. At the same time, the interpenetrating polymer network, large cross-linking degree, and the strong interaction between polyurethane molecules reduce the movement ability of the molecular segments to a certain extent, resulting in a decrease in the elongation at break and an increase in the hardness (Figure 7) of the composite.

At low temperatures, with the increase in polyurethane content, the trend of changes in the tensile strength and elongation at the break in the composite is similar to that at room temperature. When the polyurethane content reaches 3 phr, the tensile strength of MSR-3 reaches the highest value of 5.14 MPa, which is slightly lower than its performance at room temperature. Comparing the mechanical properties at low temperature and room temperature, it was found that the tensile strength and elongation at the break in the silicone rubber composite without polyurethane are almost the same, maintaining good mechanical properties. This is related to the molecular structure of silicone rubber [27,28,29,30], and the interaction force between silicone rubber molecules has little change with temperature. After adding polyurethane, the tensile strength of the composites at low temperatures is also higher than that of MSR-0. Meanwhile, the tensile strength and elongation at the break in the composites at low temperatures show little change compared to room temperature, indicating that the materials have excellent low-temperature performance and can meet the requirements of low-temperature applications.

### 3.6. Thermal Stability

**Figure 8:**
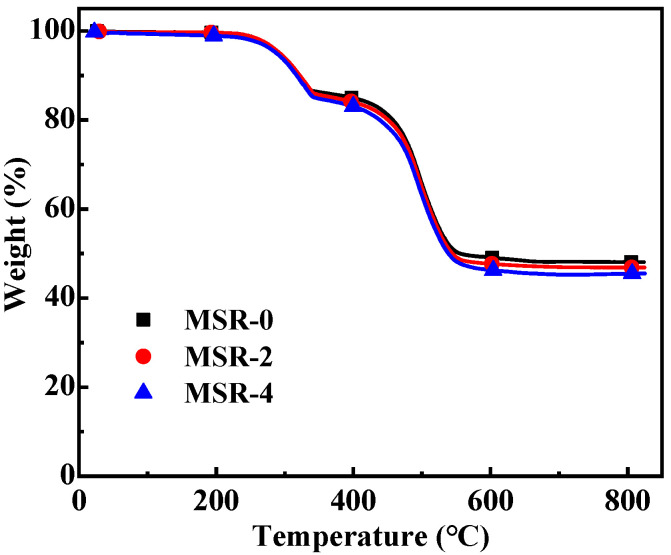
Thermogravimetric curves of silicone rubber composites.

Thermogravimetric (TG) curves of silicone rubber composites are shown in Figure 8. It can be seen that the epitaxial initial thermal decomposition temperature of all the materials is above 270 °C, exhibiting good heat resistance. The epitaxial initial thermal decomposition temperature is defined as the temperature corresponding to the intersection of the tangent line of the point with the fastest degradation rate in the first step and the baseline. There are two steps in the TG curves, so the thermal decomposition process is divided into two stages. The epitaxial initial thermal decomposition temperatures of MSR-0, MSR-2, and MSR-4 are 274.6, 275.6, and 278.1 °C, respectively (Table 2). The temperature at the end of the first stage of thermal decomposition is approximately 360 °C. With the increase in polyurethane content, the epitaxial initial thermal decomposition temperature of the material slightly increases. The first stage of weight loss is mainly caused by the dehydration of aluminum hydroxide and the thermal decomposition of the urethane bond in polyurethane. As the polyurethane content increases, its weight loss slightly increases in this stage. The decomposition temperature range of the second stage is about 460–600 °C, and the weight loss is mainly caused by the decomposition of aluminum hydroxide [31,32,33] and the break of the main chain of silicone rubber and polyurethane. Finally, the residual rate of composites after decomposition is over 45%.

### 3.7. Flame Retardant Performance

**Figure 9:**
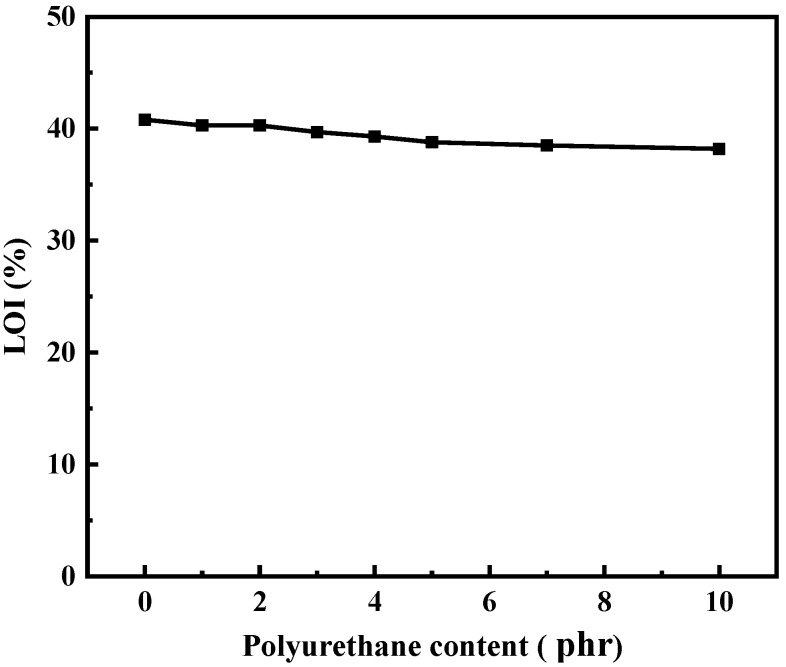
Effect of polyurethane content on the LOI of silicone rubber composites.

The limiting oxygen index (LOI) is an important index of the flame retardant material. The LOI of silicone rubber composites with different polyurethane content was tested, and the results are shown in Figure 9. The LOI value of silicone rubber composite decreases slightly with the increase in polyurethane content. Usually, an LOI greater than 27% is required for the qualification of self-extinguishing. When the polyurethane content is 10 phr, the LOI value of the composite is the lowest, which is 38.2%, much higher than 27%, so all the composites still have a good flame retardant performance.

### 3.8. Morphology of Materials

**Figure 10:**
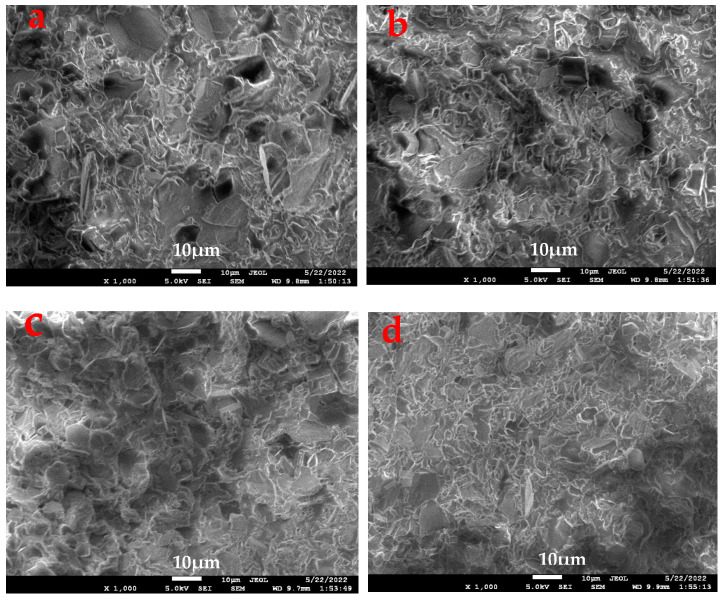
SEM images of silicone rubber composites: (**a**) MSR-0, (**b**) MSR-2, (**c**) MSR-4, and (**d**) MSR-7.

SEM images of MSR-0, MSR-2, MSR-4 and MSR-7 are shown in Figure 10. Silicone rubber forms a continuous phase in the system for MSR-0, and the phase of the interface is obvious, and the aggregation state of silicone rubber in the composite system is unevenly distributed. After introducing the polyurethane, the phase interface gradually becomes blurred, and the compatibility of the phases is improved. MSR-4 and MSR-7 have a more uniform distribution. According to the data on mechanical properties in Figure 6, it can be confirmed that the tensile strength of the materials is greatly improved because of the increase in dispersibility and compatibility after introducing the polyurethane.

It can be seen from the properties of silicone rubber composites that the mechanical properties and compatibility of the composites are increased after adding polyurethane. However, due to the presence of hydrophilic amino ester and ether bonds in the polyurethane molecules, the water contact angle of the composites decreases. Therefore, when polyurethane is used to modify silicone rubber composite, the content of polyurethane cannot be too large, and the influence of mechanical, electrical insulation and hydrophobic properties should be comprehensively considered. Of course, it is also possible to consider changing the type and structure of polyurethane, such as reducing the content of amino ester and ether bonds, in order to improve the mechanical properties of silicone rubber while maintaining high hydrophobicity as much as possible.

## 4. Conclusions

A series of modified silicone rubber composites with different polyurethane content were prepared under the condition of a fixed silicone rubber compounds proportion. After adding polyurethane, the tensile strength (at room and low temperatures), hardness, and compatibility of the silicone rubber composites are increased. However, the water absorption of the composites slightly increases, and the water contact angle decreases, thus reducing the hydrophobicity of the materials to a certain extent. Finally, considering all factors, when the polyurethane content is 3 phr, the volume resistivity, water contact angle, tensile strength and elongation at break are 19.6 PΩ·mm, 107.7°, 5.2 MPa and 889.7%, respectively, indicating that the composite has good comprehensive properties. At the same time, the composite also has good heat resistance and flame retardancy, so it can be used as an alternative material for composite sheathing arresters.

## Figures and Tables

**Table 1:** Weight loss rate of silicone rubber composites after extraction.

Composites	MSR-0	MSR-1	MSR-2	MSR-3	MSR-4	MSR-5	MSR-7	MSR-10
**Weight loss rate (%)**	0	0.001	0.001	0	0.001	0.001	0	0.001

**Table 2:** Thermal decomposition data of silicone rubber composites.

Composites	The First-Step Thermal Decomposition		The Second-Step Thermal Decomposition		Total Weight Loss Rate (%)	Residual Rate (%)
	Temperature Range (°C)	Weight Loss Rate (%)	Temperature Range (°C)	Weight Loss Rate (%)		
MSR-0	274.6–357.9	13.87	462.4–592.8	37.66	51.53	48.47
MSR-2	275.6–359.3	14.68	462.2–595.7	38.59	53.27	46.73
MSR-4	278.1–356.8	15.49	459.8–605.6	39.25	54.74	45.26

## Data Availability

Not applicable.
